# Colchicine in Patients with Acute Ischemic Stroke: A Meta-Analysis of Randomized Trials

**DOI:** 10.3390/jcm15176528

**Published:** 2026-08-24

**Authors:** Joshuan J. Barboza, Oriana Rivera-Lozada, César Bonilla-Asalde, Luis G. Chancha-Martínez, Víctor A. Mañuico-Antay, Alexis Ccoyllo-Mayhuasca, Luis E. Balcázar-Rincón, Dante Raúl Torres-Huamaní, Briseidy Ortiz-Rodríguez, Mahycol Bravo-Ramirez

**Affiliations:** 1 Vicerrectorado de Investigación, Universidad Señor de Sipán, Chiclayo 13009, Peru; riveraoriana@uss.edu.pe (O.R.-L.); bonillacesar@uss.edu.pe (C.B.-A.); 2 Facultad de Ciencias de la Salud, Universidad Científica del Sur, Lima 15047, Peru; 100076301@cientifica.edu.pe (L.G.C.-M.); 100076702@cientifica.edu.pe (V.A.M.-A.); 100080019@cientifica.edu.pe (A.C.-M.); 3 Hospital General de Zona No. 2, Instituto Mexicano del Seguro Social, Ciudad de México 06800, Mexico; umqbalcazar@gmail.com; 4 Universidad Nacional San Luis Gonzaga, Ica 11001, Peru; dante2021th@gmail.com; 5 Facultad de Ciencias de la Cultura Física, Universidad Autónoma de Chihuahua, Chihuahua 31000, Mexico; bortizr@uach.mx; 6 Tau Clinical Research Methods Network, Trujillo 13001, Peru; doctoradobravo@gmail.com

**Keywords:** colchicine, ischemic stroke, stroke, randomized controlled trials, systematic review

## Abstract

**Background:** Inflammation plays a key role in atherosclerosis and thromboinflammatory processes involved in ischemic stroke. Colchicine, an anti-inflammatory agent that inhibits microtubule polymerization and inflammasome activation, has demonstrated cardiovascular benefits in coronary artery disease. However, its effectiveness in secondary prevention after ischemic stroke remains uncertain. We conducted a systematic review and meta-analysis of randomized controlled trials (RCTs) evaluating colchicine in two pre-specified populations: (i) patients with non-cardioembolic ischemic stroke or high-risk transient ischemic attack (TIA), and (ii) atherosclerotic cardiovascular disease (ASCVD) cohorts reporting cerebrovascular outcomes, with pre-planned subgroup and sensitivity analyses restricted to stroke/TIA-only trials to address indirectness. **Methods:** We searched PubMed, Scopus, Web of Science, Embase, and the Cochrane Central Register of Controlled Trials (CENTRAL) from inception to 20 October 2025, with no language restrictions, using a fully reported strategy combining controlled vocabulary (MeSH/Emtree) and free-text terms for colchicine and cerebrovascular/atherosclerotic outcomes. The primary outcome was recurrent stroke. Secondary outcomes included composite major vascular events, serious adverse events, any adverse events, and treatment discontinuation due to adverse events. A pre-specified subgroup analysis compared stroke/TIA-dedicated trials versus ASCVD trials reporting cerebrovascular outcomes, and a sensitivity analysis was restricted to stroke/TIA-only trials. Random-effects meta-analysis was performed using risk ratios (RR) with 95% confidence intervals (CI). Heterogeneity was assessed using the I^2^ statistic, and the certainty of evidence was evaluated using the GRADE framework. **Results:** Five randomized controlled trials, including 18,336 participants, were included. Colchicine did not significantly reduce the risk of recurrent stroke compared with control therapy (RR 0.86, 95% CI 0.65–1.12). Similarly, no significant reduction in vascular events was observed (RR 0.70, 95% CI 0.40–1.23), with substantial heterogeneity across studies. Colchicine was not associated with a significant increase in serious adverse events (RR 1.14, 95% CI 0.43–2.97). When restricted to stroke/TIA-dedicated trials (CHANCE-3, CONVINCE, COPS), the point estimate for recurrent stroke remained close to the null. Across trials reporting tolerability, colchicine was associated with higher rates of gastrointestinal adverse events and study-drug discontinuation, although the magnitude varied across studies. According to GRADE, the certainty of evidence ranged from moderate for recurrent stroke to low for vascular events and serious adverse events. **Conclusions:** Colchicine did not significantly reduce recurrent stroke or vascular events in patients with ischemic stroke or transient ischemic attack. Current evidence does not support routine use of colchicine for secondary stroke prevention, and further randomized trials are needed to identify potential subgroups that may benefit from anti-inflammatory therapy.

## 1. Introduction

Stroke remains the second leading cause of death and a leading cause of disability worldwide, affecting nearly 100 million people and causing more than 7 million deaths annually [1]. Beyond the acute phase, the cumulative risk of recurrence after a first ischemic event is high, and effective secondary prevention strategies are therefore a central objective of contemporary cerebrovascular medicine [2,3,4]. Even with optimized antiplatelet therapy, lipid-lowering agents, and blood-pressure control, a substantial residual inflammatory risk persists in patients with atherosclerotic ischemic stroke or high-risk transient ischemic attack (TIA), motivating the search for novel anti-inflammatory interventions [5,6].

Vascular inflammation represents the pathophysiological gateway connecting atherosclerosis, plaque instability, and prothrombotic states with the development of ischemic stroke [7]. Atherosclerosis is now recognized as a chronic inflammatory disease characterized by endothelial dysfunction, monocyte/macrophage infiltration, and sustained activation of innate immune pathways that promote plaque growth, destabilization, and rupture, leading to arterial thrombi responsible for atherothrombotic stroke [8]. Consequently, chronic systemic inflammation also enhances platelet activation, adhesion molecule expression, and a prothrombotic state, thereby increasing the risk of cerebrovascular recurrence even in patients receiving antithrombotic therapies [9]. Although chronic inflammation may also contribute to atrial remodeling and atrial fibrillation, the present review specifically focuses on non-cardioembolic mechanisms; cardioembolic pathways are therefore not discussed further [10,11]. This immunoinflammatory framework has driven the development of therapeutic strategies targeting inflammation [12].

Colchicine has long been used as an anti-inflammatory drug for gout and, more recently, for cardiovascular inflammatory conditions such as pericarditis, facilitating its translation to vascular prevention. Its clinical appeal stems from being an inexpensive, widely available agent with a well-characterized mechanism and generally acceptable safety profile at low doses, provided that gastrointestinal tolerability and drug–drug interactions are monitored. Several randomized trials in acute coronary syndrome or stable coronary disease have shown that low-dose colchicine reduces major cardiovascular events (non-fatal myocardial infarction, non-fatal stroke, and cardiovascular death) when added to guideline-directed therapy [13]. However, in most of these trials, ischemic stroke has been included as a component of composite MACE endpoints rather than as a specific primary outcome, limiting the precision of effect estimates for this event [14].

Evidence on secondary stroke prevention remains contradictory: modest or non-significant reductions in recurrence have been observed in subgroups of patients with prior stroke or TIA, and a recent dedicated trial has even suggested a possible excess of strokes in specific high-risk phenotypes, such as symptomatic intracranial atherosclerotic stenosis in older individuals [15]. Importantly, the available trials differ markedly in the timing of colchicine initiation relative to the index event: ultra-acute initiation (within 24 h, as in CHANCE-3), subacute initiation (days to weeks, as in COPS and CONVINCE), and chronic stable disease (as in LoDoCo and LoDoCo2). Because vascular inflammation peaks early after an ischemic event and progressively declines, this temporal heterogeneity is a key driver of clinical and statistical variability and is explicitly examined in our subgroup analyses. Despite the consolidation of colchicine as an anti-inflammatory strategy in atherosclerotic disease, this heterogeneous body of evidence—particularly in cerebrovascular populations and in subgroups underrepresented in coronary trials—leaves substantial clinical uncertainty [16].

In June 2023, the United States Food and Drug Administration approved low-dose colchicine as a secondary preventive treatment for atherosclerotic disease [17]. Since then, additional large-scale, long-term randomized trials have been initiated in acute coronary syndrome and ischemic heart disease, alongside collaborative trial-level meta-analyses in established vascular disease. These analyses have refined estimates of treatment effects on myocardial infarction, stroke, and MACE and confirmed an acceptable safety profile at 0.5 mg/day [18]. Although recent meta-analyses suggest that colchicine consistently reduces ischemic stroke and MACE in atherosclerotic disease, these analyses pool coronary and cerebrovascular populations and do not specifically focus on recent-onset non-cardioembolic stroke, making it difficult to precisely define the benefit–risk balance in this clinical window [19]. A meta-analysis dedicated to colchicine in non-cardioembolic ischemic stroke or high-risk TIA—and explicitly addressing the indirectness introduced by coronary trials reporting stroke as part of composite endpoints—is therefore warranted.

The aim of this systematic review and meta-analysis was to evaluate the efficacy and safety of low-dose colchicine compared with placebo or usual care in two pre-specified populations: (i) adult patients with non-cardioembolic acute ischemic stroke or high-risk TIA; and (ii) ASCVD cohorts reporting cerebrovascular outcomes. Within this framework, we additionally performed pre-planned subgroup and sensitivity analyses restricted to stroke/TIA-only trials, and explored the effect of timing of colchicine initiation on the observed treatment effects.

## 2. Materials and Methods

This systematic review and meta-analysis was designed and reported in accordance with the Preferred Reporting Items for Systematic Reviews and Meta-Analyses (PRISMA) 2020 statement. The completed PRISMA 2020 checklist is provided as Supplementary Table S1. The review was not prospectively registered in PROSPERO, and a formal a priori protocol was not published; however, the eligibility criteria, outcomes, and all pre-planned subgroup and sensitivity analyses were specified before the screening and data extraction phases, as detailed in Section 2.1 and Section 2.6.

### 2.1. Eligibility Criteria

Eligibility was defined using the PICOS framework. (1) Study design: Published, peer-reviewed parallel-group randomized controlled trials (RCTs). (2) Population: Adults (≥18 years) belonging to one of two pre-specified strata—(a) non-cardioembolic acute ischemic stroke or high-risk TIA (stroke/TIA stratum: CHANCE-3, CONVINCE, COPS); or (b) atherosclerotic cardiovascular disease cohorts (LoDoCo, LoDoCo2) in which recurrent stroke and/or composite cerebrovascular outcomes were pre-specified and adjudicated. The two strata are analyzed jointly and separately to address the indirectness highlighted by the reviewers. (3) Intervention: Colchicine at any oral dose. (4) Comparator: Placebo or usual care. (5) Outcomes: At least one of recurrent stroke, composite vascular events, serious adverse events, any adverse events, or treatment discontinuation due to adverse events. Systematic reviews, meta-analyses, narrative reviews, editorials, conference abstracts, case reports, case series, non-randomized studies, and single-arm studies were excluded.

### 2.2. Information Sources and Search Strategy

A systematic search was conducted in PubMed/MEDLINE, Scopus, Web of Science, Embase, and the Cochrane Central Register of Controlled Trials (CENTRAL) from database inception to 20 October 2025, with no language restrictions. The search combined controlled vocabulary (MeSH and Emtree) and free-text terms for the intervention and the population, structured around the following blocks: (i) intervention—“colchicine” [MeSH], colchicine, low-dose colchicine, anti-inflammatory; (ii) cerebrovascular outcomes—“stroke” [MeSH], ischemic stroke, acute ischemic stroke, transient ischemic attack, TIA, cerebrovascular accident, recurrent stroke, non-cardioembolic stroke, intracranial atherosclerosis; (iii) atherosclerotic cardiovascular disease—atherosclerotic cardiovascular disease, coronary artery disease, acute coronary syndrome, secondary prevention; and (iv) trial filter—randomized controlled trial[pt], randomized, randomised, placebo. Blocks (i) AND [(ii) OR (iii)] AND (iv) were combined with Boolean operators and adapted for each database (e.g., Emtree terms in Embase; TS= operators in Web of Science). Reference lists of included studies and relevant reviews were hand-searched, and ClinicalTrials.gov was screened for completed but unpublished trials. The full database-specific search strategies, including all synonyms, MeSH/Emtree descriptors, date limits (inception–20 October 2025), and the absence of language restrictions, are reported in Supplementary Table S2. Two reviewers (L.G.C.-M. and V.A.M.-A.) independently executed the searches; the results were de-duplicated and managed in Rayyan QCRI.

### 2.3. Study Selection

An electronic literature search was conducted, and the retrieved records were compiled into a single database by three authors (L.E.B.-R., D.R.T.-H. and B.O.-R.). The selection process was carried out using Rayyan QCRI (https://rayyan.qcri.org/, last accessed: 22 November 2025). After removing duplicates, three reviewers (L.G.C.-M., V.A.M.-A. and A.C.-M.) independently assessed titles and abstracts. Full texts of potentially eligible records were screened against the PICOS criteria. Discrepancies were resolved by consensus, with arbitration by a senior reviewer (J.J.B.) when needed [20].

### 2.4. Data Extraction

For each eligible study, data were extracted independently using a standardized form. The extracted information included author details, year of publication, study design, country, sample size for each intervention and control group, eligibility criteria, characteristics of the interventions and comparators, timing of colchicine initiation relative to the index event (ultra-acute < 24 h, subacute days–weeks, or chronic stable disease), duration of follow-up, and primary and secondary outcomes, including counts of recurrent stroke, composite vascular events, serious adverse events, any adverse events, and study-drug discontinuation due to adverse events when reported. A third reviewer independently verified the accuracy and completeness of the extracted data.

### 2.5. Risk of Bias Assessment

Risk of bias was assessed using the revised Cochrane Risk of Bias (RoB 2) tool for RCTs [21]. Each outcome was scored as “low risk”, “some concerns”, or “high risk”. The overall risk of bias was rated as low if all domains were at low risk, some concerns if at least one domain raised some concerns but none was at high risk, and high risk if any domain was at high risk or if multiple domains raised some concerns. Two authors (M.B.-R. and O.R.-L.) performed the assessment independently; discrepancies were resolved by discussion with a third author (J.J.B.).

### 2.6. Meta-Analysis

Meta-analyses were performed using the inverse-variance method with a random-effects model. Effects for dichotomous outcomes were expressed as risk ratios (RR) with 95% confidence intervals (CI) computed by the Hartung–Knapp–Sidik–Jonkman (HKSJ) method, with τ^2^ estimated by the Paule–Mandel approach. Heterogeneity was quantified by the I^2^ index (low ≤ 30%, moderate 30–60%, high > 60%) and complemented by 95% prediction intervals. To address the heterogeneity and indirectness raised by the reviewers, the following pre-planned analyses were undertaken: (i) a primary analysis including all eligible RCTs; (ii) a sensitivity analysis restricted to stroke/TIA-dedicated trials (CHANCE-3, CONVINCE, COPS); (iii) a subgroup analysis stratified by timing of colchicine initiation (ultra-acute < 24 h, subacute days–weeks, chronic stable disease); and (iv) an exploration of the influence of individual trials by leave-one-out analysis. Forest plots were produced for all primary and secondary outcomes. A two-sided *p*-value < 0.05 was considered statistically significant. Analyses were conducted in R version 4.5.2 (R Foundation for Statistical Computing) using the meta and metafor packages.

### 2.7. Assessment of the Certainty of the Evidence

The certainty of the evidence for each outcome was assessed using the GRADE framework, evaluating risk of bias, imprecision, indirectness (with explicit consideration of the inclusion of ASCVD cohorts), inconsistency, and publication bias [22]. Indirectness was specifically downgraded when most of the weight came from coronary trials with stroke as a component of composite endpoints.

## 3. Results

### 3.1. Study Selection

**Figure 1:**
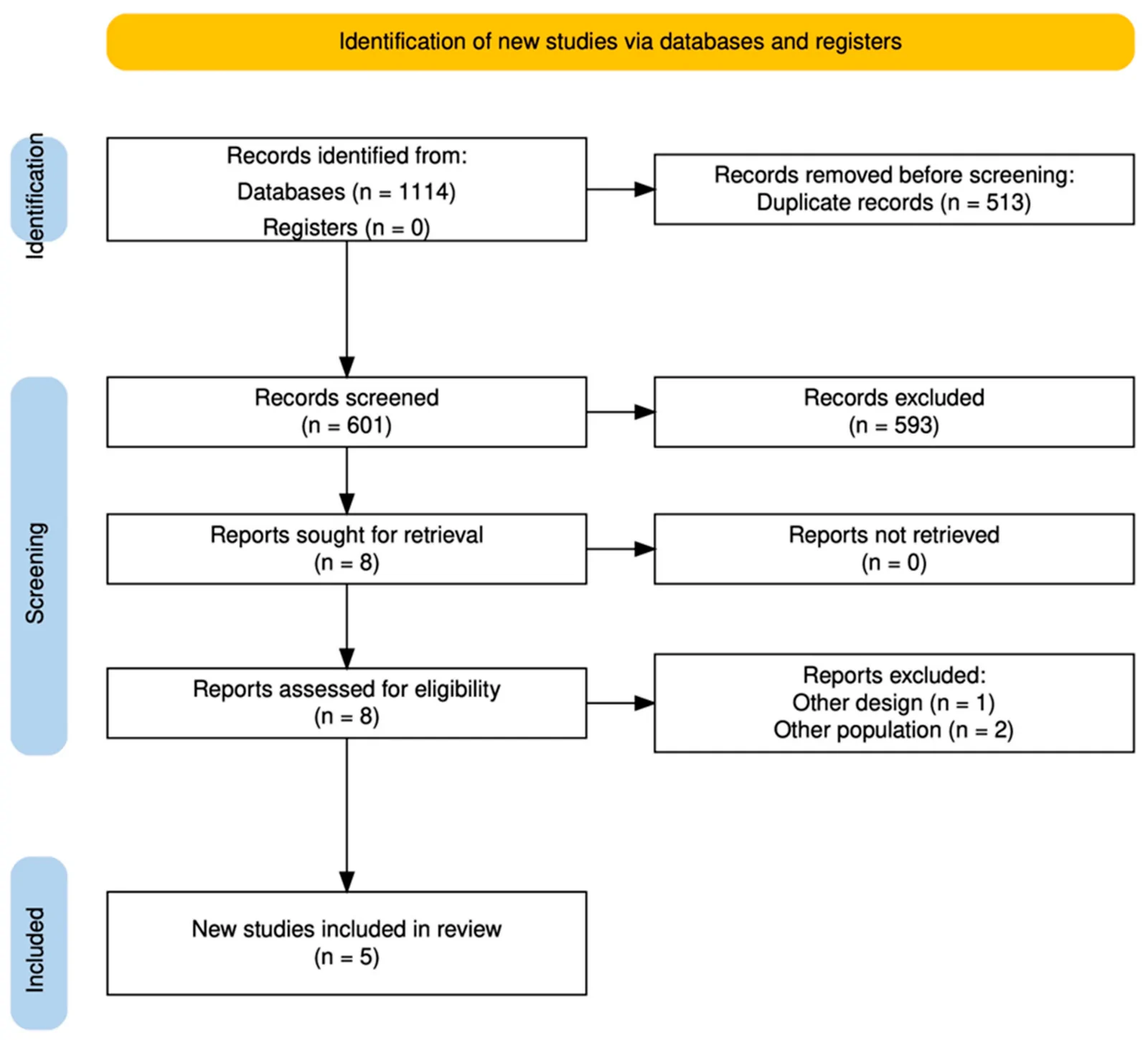
PRISMA 2020 flow chart.

The literature search identified 1114 records from electronic databases. The selection process is illustrated in the PRISMA 2020 flow diagram (Figure 1). Following the removal of 513 duplicates, 601 records were screened at the title and abstract levels, resulting in the exclusion of 593 records. Eight full-text articles were assessed for eligibility; three were excluded owing to inappropriate study design or non-eligible populations (Supplementary Table S3). Finally, five studies met the inclusion criteria and were included in the systematic review and meta-analysis [23,24,25,26,27].

### 3.2. Characteristics of the Included Studies

Of the five included studies, two were conducted exclusively in Australia, one included populations from Australia and the Netherlands, one in China, and one included European countries and Canada over the last two decades. The total number of participants was 18,336 [23,24,25,26,27]. All five RCTs used low-dose colchicine (0.5 mg/day) as the maintenance dose, although the timing of initiation differed markedly: ultra-acute (<24 h after symptom onset) in CHANCE-3, subacute (days–weeks) in COPS and CONVINCE, and chronic stable disease in LoDoCo and LoDoCo2, compared with placebo or usual care. The uniformity of the maintenance dose facilitated comparability between studies, despite differences in treatment duration and initial loading regimens. Across trials, the individual study conclusions were heterogeneous: LoDoCo, LoDoCo2 and COLCOT reported reductions in composite cardiovascular events in ASCVD populations; CHANCE-3 and CONVINCE did not show a significant reduction in recurrent stroke in dedicated cerebrovascular cohorts. These differences in study population, timing of initiation and outcome definitions (Table 1) are central to the interpretation of the pooled estimates reported below.

### 3.3. Risk of Bias Assessment

Across the included randomized trials, the risk of bias was generally low in outcome measurement domains because clinical endpoints were objective and frequently adjudicated by blinded committees (Table 2). Trials using placebo-controlled, double-blind designs (CHANCE-3 and LoDoCo2) were judged to be at low overall risk of bias. In contrast, trials without placebo (CONVINCE and LoDoCo) raised concerns about potential performance-related deviations from the intended interventions, despite masked endpoint adjudication and intention-to-treat analyses. For COPS, some concerns were primarily driven by incomplete outcome data (attrition), whereas other domains were assessed as low risk.

### 3.4. Effect of Colchicine on Primary Outcomes

#### 3.4.1. Recurrent Stroke

**Figure 2:**
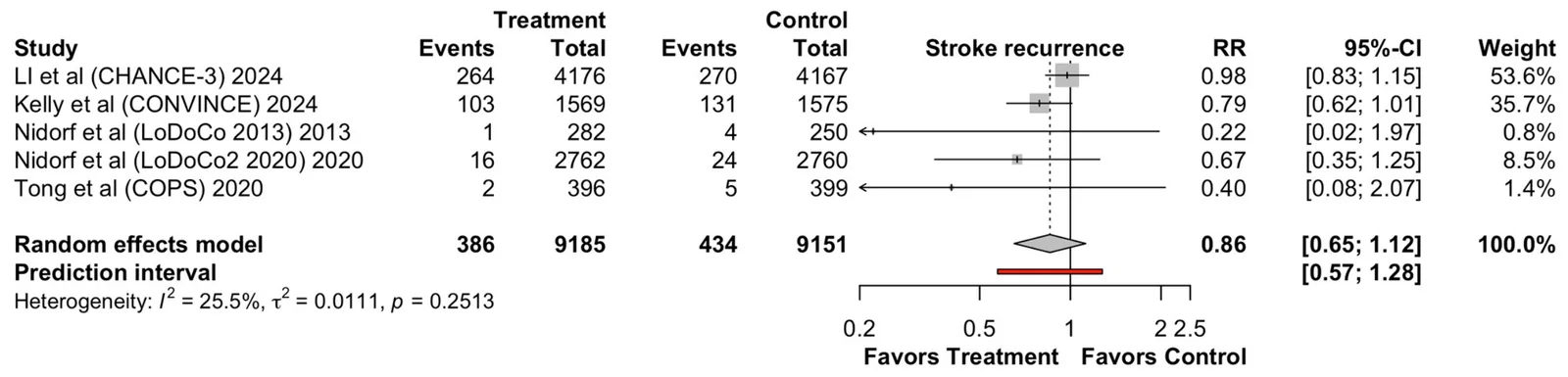
Effect of colchicine on stroke recurrence.

The pooled analysis of five RCTs (18,336 participants) evaluated the effect of colchicine on recurrent stroke (Figure 2). Stroke recurrence occurred in 386/9185 participants in the colchicine group and 434/9151 in the control group. The random-effects meta-analysis showed no statistically significant reduction in recurrent stroke (RR 0.86, 95% CI 0.65–1.12). Between-study heterogeneity was low to moderate (I^2^ = 25.5%, τ^2^ = 0.0111, *p* = 0.2513). Individually, the largest trials (CHANCE-3 and CONVINCE) contributed most of the statistical weight and reported effects close to the null, whereas smaller trials (LoDoCo, COPS) showed greater variability with wide confidence intervals. The prediction interval ranged from 0.57 to 1.28. In the pre-planned sensitivity analysis restricted to stroke/TIA-dedicated trials (CHANCE-3, CONVINCE, COPS), the pooled effect remained close to the null and non-significant, with markedly reduced heterogeneity, indicating that the modest point-estimate benefit observed in the primary analysis was largely driven by ASCVD trials in which stroke was a secondary component. Accordingly, although Section 3.2 summarizes the individual study conclusions (some of which favored colchicine), the pooled estimate does not demonstrate a statistically significant reduction in recurrent stroke.

#### 3.4.2. Composite Vascular Events

**Figure 3:**
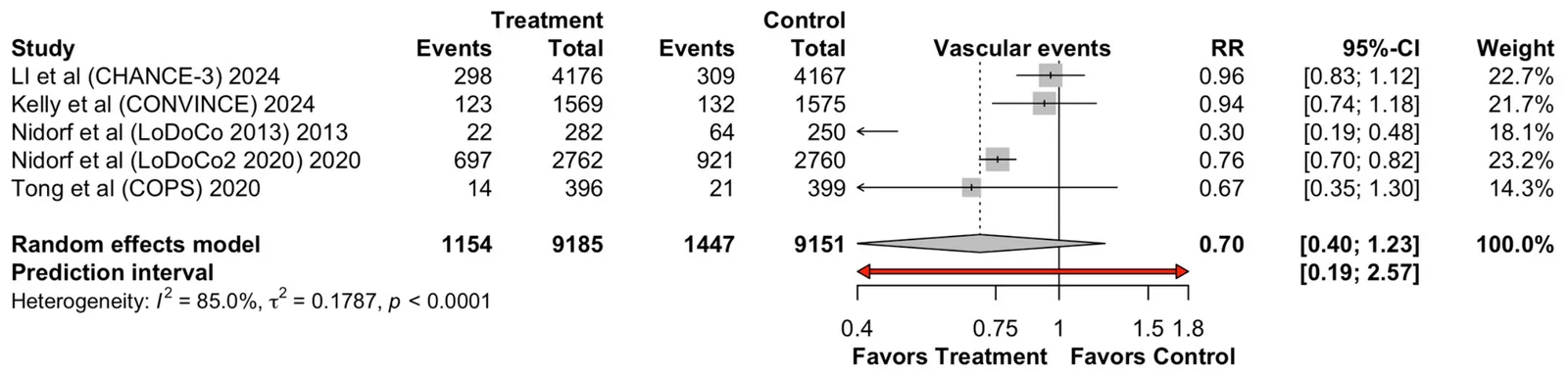
Effects of colchicine on vascular events.

Five RCTs evaluated the composite outcome of major vascular events (Figure 3). A total of 1154 events occurred among 9185 participants in the colchicine group and 1447 events among 9151 in the control group. The random-effects model suggested a potential reduction (RR 0.70), with the confidence interval crossing the null (95% CI 0.40–1.23). Substantial heterogeneity was observed (I^2^ = 85.0%, τ^2^ = 0.1787, *p* < 0.0001). LoDoCo2 contributed most of the protective signal, whereas stroke-dedicated trials showed more neutral estimates. The prediction interval was wide (0.19–2.57). Because the composite endpoint differed substantially across trials (e.g., MACE in LoDoCo2 vs. recurrent ischemic events in CHANCE-3 and CONVINCE), this outcome must be interpreted with caution; results were robust in leave-one-out analyses except when LoDoCo2 was excluded, after which the pooled estimate moved towards the null. This pattern, together with the indirectness of the composite outcome, was the basis for the GRADE downgrade.

#### 3.4.3. Serious Adverse Events

**Figure 4:**
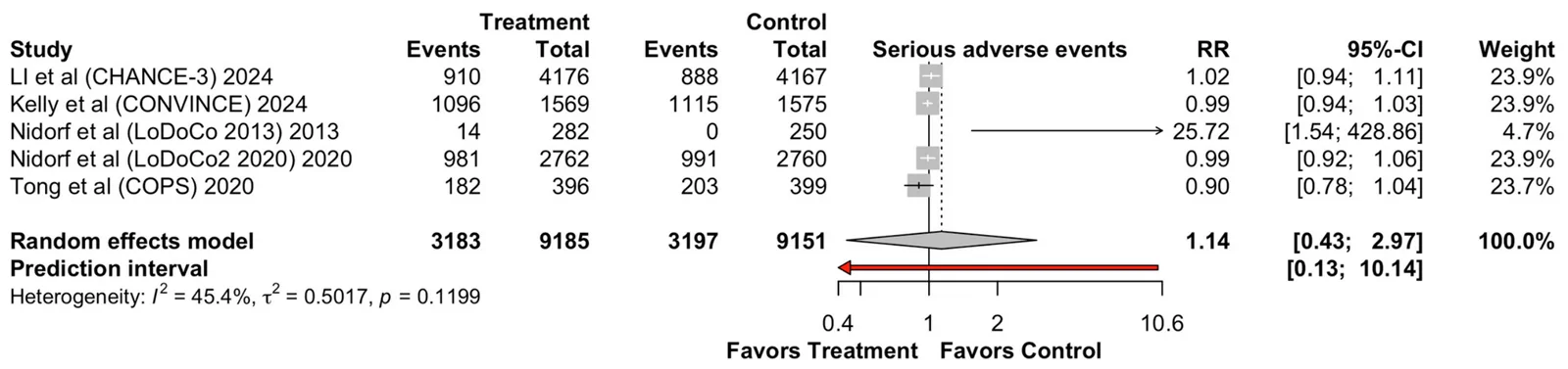
Effects of colchicine on serious adverse events.

Serious adverse events (SAEs) were reported in all five trials (Figure 4). A total of 3183 events occurred among 9185 participants in the colchicine group and 3197 events among 9151 in the control group. The pooled random-effects analysis showed no statistically significant difference (RR 1.14, 95% CI 0.43–2.97). Moderate heterogeneity was observed (I^2^ = 45.4%, τ^2^ = 0.5017, *p* = 0.1199). Most trials reported risk estimates close to unity, suggesting comparable safety profiles. The original LoDoCo [23] trial reported markedly fewer cumulative SAEs than the larger contemporary trials because, as an open-label pilot in stable coronary disease, it pre-specified a narrower SAE definition focused on prompt hospitalization for serious cardiovascular or gastrointestinal events, did not include a placebo arm, and used active follow-up to capture events. The apparent zero-event count in the comparator group therefore reflects a different operational SAE definition and a shorter event-capture window rather than an implausible safety profile; for this reason, a continuity correction was applied, and the inclusion of LoDoCo was examined in the leave-one-out analysis. The prediction interval was very wide (0.13–10.14), highlighting uncertainty in the safety estimate. Overall, low-dose colchicine did not increase the incidence of SAEs in the pooled analysis. Nevertheless, beyond SAEs, the included trials consistently reported higher rates of gastrointestinal adverse events with colchicine (predominantly diarrhea and nausea); rates of any adverse events and of study-drug discontinuation due to adverse events were higher in the colchicine arms across CHANCE-3, CONVINCE and COPS, and ranged from approximately 5% to 15% depending on the trial. This tolerability profile is clinically relevant, as it may attenuate intention-to-treat efficacy in practice and is discussed in Section 4.1 (Safety considerations).

### 3.5. Certainty of the Evidence (GRADE)

The certainty of the evidence for each outcome was assessed using the GRADE framework. Results are summarized in Table 3. For recurrent stroke, certainty was rated moderate, downgraded for imprecision (confidence interval crossing the null and wide prediction interval). For composite vascular events, certainty was rated low, downgraded for serious inconsistency (I^2^ = 85%) and imprecision, and additionally considering indirectness from the inclusion of ASCVD trials with heterogeneous composite definitions. For SAEs, certainty was rated low, downgraded for very serious imprecision.

**Table 1:** Characteristics of the included studies.

Authors	Year	Country/Region	Sample/ Participants	Key Inclusion Criteria	Type of Intervention	Type of Control	Primary Outcome	Secondary Outcome
Nidorf et al. [23]	2013	Australia	532 patients	Chronic stable coronary disease for at least 6 months before enrollment.	Colchicine 0.5 mg once daily	No colchicine	Noncardioembolic stroke (n,%): Colchicine 1/282 (0.35%) No Colchicine 4/250 (1.6%)	Vascular Events: Angina, acute coronary syndrome, vascular death (n,%): Colchicine 22/282 (7.8%) No Colchicine 64/250 (25.6%)
Tong et al. [24]	2020	Australia	795 patients	Chronic coronary artery disease and evidence of coronary inflammation	Colchicine 0.5 mg twice daily for one month, followed by 0.5 mg once daily	Placebo	Noncardioembolic stroke (n,%): Colchicine 2/396 (0.50%) Placebo 6/399 (1.5%)	Vascular Events: Angina, acute coronary syndrome, vascular death (n,%): Colchicine 14/396 (3.53%) Placebo 21/399 (5.26%)
Nidorf et al. [25]	2020	Australia/Netherlands	5522 patients	Evidence of Chronic coronary disease on invasive angiography or computed tomography angiography or a coronary calcium score of ≥400 Agatston units on a coronary artery calcium scan; clinically stable condition for ≥6 months before enrollment.	Colchicine 0.5 mg once daily	Placebo	Noncardioembolic stroke (n,%): Colchicine 16/2762 (0.57%) Placebo 24/2760 (0.86%)	Vascular Events: Angina, acute coronary syndrome, vascular death (n,%): Colchicine 697/2762 (25.23%) Placebo 921/2760 (33.36%)
Li et al. [26]	2024	China	8343 patients	Patients aged 40 years or older with an acute minor-to-moderate ischaemic stroke (NIHSS score ≤ 5) or a high-risk transient ischaemic attack (ABCD^2^ score ≥ 4) and a baseline high-sensitivity C-reactive protein (hs-CRP) level ≥ 2 mg/L	Colchicine 0.5 mg twice daily on days 1–3, followed by 0.5 mg daily from days 4 to 90.	Placebo	Any new stroke (ischaemic or haemorrhagic) within 90 days (n,%): Colchicine: 264/4176 (6.3%). Placebo: 270/4167 (6.5%).	Composite vascular events (stroke, TIA, MI, or vascular death) (n,%): Colchicine: 298/4176 (7.1%). Placebo: 309/4167 (7.4%)
Kelly et al. [27]	2024	13 European countries and Canada	3144 patients	Patients aged 40 years or older with non-severe ischaemic stroke (mRS ≤ 3) or high-risk TIA likely caused by atherosclerosis, lacunar disease, or cryptogenic embolism. Participants were randomised between 72 h and 28 days after the qualifying event.	Long-term colchicine (0.5 mg orally per day) plus guideline-based usual care	Usual care alone	Composite of first recurrent non-fatal ischaemic stroke, MI, cardiac arrest, hospitalisation for unstable angina, or vascular death (n,%): Colchicine: 153/1569 (9.8%). Usual care: 185/1575 (11.7%).	Key secondary endpoint (non-fatal recurrent ischaemic stroke, MI, cardiac arrest, or vascular death) (n,%): Colchicine: 147/1569 (9.4%). Usual care: 177/1575 (11.2%)

**Table 2:** Risk of bias assessment.

Study (Trial)	Year	Design Features (as Reported)	D1 Randomization	D2 Deviations	D3 Missing Data	D4 Outcome Measurement	D5 Selective Reporting	Overall RoB
Li et al. [26] (CHANCE-3)	2024	Multicenter, randomized, double-blind, placebo-controlled; masked participants/caregivers/outcome assessors; ITT analyses; adjudicated endpoints	**Low**	**Low**	**Low**	**Low**	**Low**	**Low**
Kelly et al. [27] (CONVINCE)	2024	Randomized trial; no placebo due to feasibility; web-based allocation; blinded endpoint adjudication; ITT framework; some losses/withdrawals	**Low**	**Some concerns**	**Low**	**Low**	**Low**	**Some concerns**
Nidorf et al. [23] (LoDoCo)	2013	Randomized PROBE (observer-blinded endpoint) design; no placebo; adjudicator blinded; ITT analyses	**Some concerns**	**Some concerns**	**Low**	**Low**	**Some concerns**	**Some concerns**
Nidorf et al. [25] (LoDoCo2)	2020	Randomized, controlled, double-blind, placebo-controlled; endpoint adjudication; minimal loss to follow-up reported	**Low**	**Low**	**Low**	**Low**	**Low**	**Low**
Tong et al. [24] (COPS)	2020	Multicenter, randomized, double-blind, placebo-controlled; blinded adjudication; follow-up with some attrition noted	**Low**	**Low**	**Some concerns**	**Low**	**Low**	**Some concerns**

**Table 3:** GRADE Certainty of evidence.

Outcome	No. of Studies	Study Design	Risk of Bias	Inconsistency	Indirectness	Imprecision	Publication Bias	Overall Certainty
Stroke recurrence	5 RCTs	Randomized controlled trials	Not serious	**Not serious**	**Not serious**	**Serious**	**Undetected**	**Moderate**
Vascular events	5 RCTs	Randomized controlled trials	Not serious	**Serious**	**Not serious**	**Serious**	**Undetected**	**Low**
Serious adverse events	5 RCTs	Randomized controlled trials	Not serious	**Not serious**	**Not serious**	**Very serious**	**Undetected**	**Low**

## 4. Discussion

In this systematic review and meta-analysis of randomized controlled trials including more than 18,000 participants, colchicine did not significantly reduce the risk of recurrent stroke in patients with non-cardioembolic ischemic stroke or high-risk TIA. Although the pooled estimate suggested a trend toward benefit, the confidence interval crossed the null value, and the prediction interval indicated uncertainty regarding the magnitude and direction of the effect. Similarly, the analysis of vascular events did not demonstrate a statistically significant reduction and showed substantial heterogeneity. Colchicine was not associated with a significant increase in SAEs, but tolerability—particularly gastrointestinal—was less favorable in the active arms. Overall, the evidence was rated as moderate to low according to GRADE.

Inflammation plays a fundamental role in the pathophysiology of atherosclerosis and thrombotic vascular events. Activation of the NLRP3 inflammasome and subsequent release of proinflammatory cytokines such as interleukin-1β and interleukin-6 contribute to plaque destabilization and endothelial dysfunction. Colchicine exerts anti-inflammatory effects by inhibiting microtubule polymerization, suppressing neutrophil activation, and modulating inflammasome signaling pathways. These mechanisms have been associated with reduced vascular inflammation and plaque stabilization, providing a strong biological rationale for its use in secondary prevention of atherosclerotic disease [28]. However, the cerebrovascular and coronary circulations differ in several key respects that may modulate this rationale. Coronary atherosclerosis is dominated by lipid-rich plaque rupture in epicardial arteries, whereas a substantial proportion of ischemic strokes arises from small-vessel disease, intracranial atherosclerosis, or aorto-cervical sources with distinct histopathological and inflammatory profiles. Furthermore, the blood–brain barrier limits the access of systemic inflammatory mediators and drugs to the central nervous system, and post-stroke neuroinflammation involves microglial activation and resident immune cells that respond differently to colchicine compared with peripheral neutrophils. These pathophysiological differences may explain, at least in part, the discordant magnitude of benefit observed in coronary versus cerebrovascular trials.

Among the trials that contributed most to the statistical weight of our analysis, two stroke-dedicated RCTs deserve specific attention. CHANCE-3 was a large, multicenter, double-blind, placebo-controlled trial of 8343 Chinese patients with minor ischemic stroke or high-risk TIA, in which colchicine was started within 24 h of symptom onset; no significant reduction in recurrent stroke was observed at 90 days, although post hoc analyses suggested potential benefit in subgroups defined by inflammatory biomarkers such as mean corpuscular volume and triglyceride levels [24]. CONVINCE was an open-label, blinded-endpoint trial of long-term colchicine for prevention of vascular recurrence after non-cardioembolic stroke or TIA; the trial was prematurely closed due to the COVID-19 pandemic, and the intention-to-treat analysis did not demonstrate a statistically significant reduction in the primary composite outcome, although per-protocol analyses suggested a possible benefit [25]. Both trials therefore align with our pooled estimate of a non-significant trend towards benefit, and underline that timing of initiation, inflammatory phenotype, and treatment adherence may be more important determinants of efficacy in cerebrovascular populations than in coronary populations.

Several large cardiovascular trials have reported reductions in major adverse cardiovascular events with low-dose colchicine therapy. LoDoCo and LoDoCo2 showed that colchicine significantly reduced cardiovascular events in patients with chronic coronary disease [29]. The COLCOT trial reported a reduction in ischemic cardiovascular outcomes after recent myocardial infarction [30]. In our analysis, these trials, together with their long-term post hoc analyses, contributed most of the apparent benefit observed for composite vascular endpoints; however, in these cohorts stroke was reported as part of a broader MACE composite rather than as a primary cerebrovascular outcome, which introduces indirectness when extrapolating their results to acute cerebrovascular populations. These findings have strengthened the hypothesis that targeting vascular inflammation may improve outcomes in atherosclerotic disease.

Despite the robust cardiovascular evidence, our findings suggest that the benefits of colchicine may be less pronounced in the context of secondary stroke prevention. Previous observational and pooled analyses in established ASCVD have suggested that colchicine may reduce the risk of ischemic stroke as part of composite cardiovascular outcomes [31,32]. However, most of these studies were conducted in coronary populations rather than in cohorts specifically presenting with acute ischemic stroke, and were therefore not designed to detect cerebrovascular-specific effects.

Stroke represents a heterogeneous syndrome with multiple etiological mechanisms, including large-artery atherosclerosis, cardioembolism, and small-vessel disease. Consequently, the relative contribution of vascular inflammation may differ across stroke subtypes, potentially attenuating the effect of anti-inflammatory therapies. Moreover, many participants in the included trials received contemporary evidence-based secondary prevention (statins, antiplatelet agents, intensive risk-factor control), which may have reduced the incremental benefit of colchicine.

Another factor contributing to the observed variability is the timing of colchicine initiation after the index event. In COLCOT, colchicine was started early after myocardial infarction during an intense inflammatory phase [30]. In stroke trials, CHANCE-3 started colchicine within 24 h of stroke onset, capturing the early inflammatory surge, whereas COPS and CONVINCE initiated treatment days to weeks after the event, when inflammatory biomarkers have already begun to decline, and LoDoCo/LoDoCo2 enrolled patients with chronic stable coronary disease far from any acute event. Because vascular inflammation peaks early and declines progressively, this temporal mismatch is likely a major driver of the observed heterogeneity and was therefore explored in our pre-planned subgroup analysis. Stroke trials may thus have included patients at different stages of the inflammatory cascade, potentially influencing treatment response.

### 4.1. Safety Considerations

The present meta-analysis found no significant increase in serious adverse events with colchicine therapy. This is consistent with prior randomized trials and meta-analyses in cardiovascular populations, where low-dose colchicine has generally demonstrated an acceptable safety profile. Nevertheless, colchicine has a narrow therapeutic window and is associated with frequent gastrointestinal adverse events—predominantly diarrhea and nausea—that lead to non-trivial rates of treatment discontinuation. Across the included stroke/TIA trials, study-drug discontinuation due to adverse events was higher in the colchicine arms than in the control arms (e.g., approximately 6–8% in CHANCE-3 and a higher proportion in CONVINCE and COPS), and rates of any adverse events were systematically higher than placebo. Reporting SAEs alone therefore underestimates the clinical impact of intolerance: in pragmatic intention-to-treat terms, patients who discontinue due to gastrointestinal symptoms may not derive the full anti-inflammatory benefit, which is particularly relevant when interpreting modest effect sizes in cerebrovascular populations. Colchicine use has also been associated, in some studies, with a potential increase in non-cardiovascular mortality, although this association remains controversial and may reflect chance or residual confounding [31].

The wide prediction interval observed for serious adverse events underscores the uncertainty of the safety estimate and supports the need for additional large trials specifically designed to evaluate safety and tolerability outcomes in cerebrovascular populations [33].

### 4.2. Clinical Implications

The findings of this meta-analysis suggest that routine use of colchicine for secondary prevention after ischemic stroke cannot currently be recommended. Although anti-inflammatory therapy represents a promising strategy in atherosclerotic disease, the present data indicate that its benefits may not translate uniformly across vascular territories. Future research should focus on identifying patient subgroups who may derive greater benefit from colchicine—particularly those with large-artery atherosclerosis, intracranial atherosclerotic stenosis, or evidence of heightened inflammatory activity (e.g., elevated high-sensitivity C-reactive protein, neutrophil-to-lymphocyte ratio, mean corpuscular volume or triglycerides).

### 4.3. Strengths and Limitations

This study has several strengths. We included only RCTs, used a large cumulative sample size, applied robust statistical methods (random-effects modeling with HKSJ confidence intervals, prediction intervals, and leave-one-out analyses), and assessed the certainty of evidence with GRADE. We also pre-specified separate strata for stroke/TIA trials and ASCVD trials, and conducted sensitivity and subgroup analyses to address the indirectness and temporal heterogeneity of the included evidence base.

Several limitations should be acknowledged. First, the conceptual scope of the review extends to ASCVD cohorts in which stroke is a component of composite endpoints; although this was addressed by pre-planned subgroup analyses restricted to stroke/TIA trials, residual indirectness remains. Second, the number of RCTs available for stroke populations is still limited, and heterogeneity was substantial for some outcomes. Differences in study populations, dosing regimens, follow-up duration, timing of initiation, and outcome definitions likely contributed to variability. Subgroup analyses by stroke etiology could not be performed due to insufficient individual-patient data. Third, the definition of SAEs differed across trials (e.g., LoDoCo used a narrower, hospitalization-based definition compared with CHANCE-3, CONVINCE, LoDoCo2 and COPS), which influenced both the absolute counts and the pooled estimate; this is explicitly discussed in Section 3.4.3.

Future RCTs should explore colchicine in more homogeneous cerebrovascular populations—particularly patients with large-artery atherosclerosis or intracranial atherosclerotic stenosis or elevated inflammatory biomarkers—and clarify the optimal timing and duration of therapy after acute stroke. Individual-patient-data meta-analyses and head-to-head comparisons of colchicine with emerging anti-inflammatory agents (e.g., IL-1β or IL-6 inhibitors) are also warranted.

## 5. Conclusions

In this systematic review and meta-analysis of randomized controlled trials, colchicine therapy was not associated with a statistically significant reduction in recurrent stroke among patients with acute ischemic stroke or transient ischemic attack. Although the pooled estimates suggested a possible protective effect, the confidence intervals crossed the line of no effect, and the prediction intervals indicated considerable uncertainty regarding the magnitude and direction of the treatment effect.

Similarly, colchicine did not demonstrate a significant reduction in composite vascular events, and substantial heterogeneity was observed across studies. In terms of safety, colchicine was not associated with a clear increase in serious adverse events; however, the available evidence remains imprecise.

Overall, the evidence was moderate to low, mainly due to imprecision and heterogeneity across the included trials. While colchicine has shown cardiovascular benefits in coronary artery disease populations, the current evidence does not support its routine use for secondary prevention after ischemic stroke.

Further large-scale randomized trials focusing on more homogeneous stroke populations and evaluating optimal timing, dosing strategies, and inflammatory risk profiles are needed to clarify the potential role of colchicine in secondary stroke prevention.

## Data Availability

The original contributions presented in this study are included in the article/Supplementary Material. Further inquiries can be directed to the corresponding author.

## Supplementary Materials

The following supporting information can be downloaded at https://www.mdpi.com/article/10.3390/jcm15176528/s1: Table S1. PRISMA 2020 checklist; Table S2. Search strategy for each database; Table S3. Reason for exclusion of studies.
