# Supplementary material for: Colchicine in Patients with Acute Ischemic Stroke: A Meta-Analysis of Randomized Trials
Source: J Clin Med. 2026 Aug 24;15(17):6528. doi: 10.3390/jcm15176528 (PMC13566465; doi:10.3390/jcm15176528)
Supplement: Supplementary file 1 [file jcm-15-06528-s001.zip › jcm-4310303-supplementary.pdf]

SUPPLEMENTARY MATERIAL

Supplementary table S1. PRISMA 2020 checklist……………………………………………….. 2
Supplementary table S2. Search strategy for each database………………………………. 6
Supplementary table S3. Reason for exclusion of studies…………………………………... 7

Supplementary Table S1. PRISMA 2020 Checklist

Manuscript: Colchicine in patients with acute ischemic stroke: a meta-analysis of randomized trials. (JCM-4310303, revised version).

Section and Topic	Item #	Checklist item	Location where item is reported
**TITLE**			
**Title**	1	Identify the report as a systematic review.	Title page (article type: "Systematic review"; title includes "meta-analysis of randomized trials").
**ABSTRACT**			
**Abstract**	2	See the PRISMA 2020 for Abstracts checklist.	Abstract (Background, Methods, Results, Conclusions).
**INTRODUCTION**			
**Rationale**	3	Describe the rationale for the review in the context of existing knowledge.	Section 1. Introduction (paragraphs 1–4).
**Objectives**	4	Provide an explicit statement of the objective(s) or question(s) the review addresses.	Section 1. Introduction (last paragraph: aim of the review).
**METHODS**			
**Eligibility criteria**	5	Specify the inclusion and exclusion criteria for the review and how studies were grouped for the syntheses.	Section 2.1. Eligibility Criteria (PICOS framework).
**Information sources**	6	Specify all databases, registers, websites, organisations, reference lists and other sources searched or consulted to identify studies. Specify the date when each source was last searched or consulted.	Section 2.2. Information Sources and Search Strategy (PubMed/MEDLINE, Scopus, Web of Science, Embase, CENTRAL; from inception to 20 October 2025). Supplementary Table S2.
**Search strategy**	7	Present the full search strategies for all databases, registers and websites, including any filters and limits used.	Section 2.2 and Supplementary Table S2 (full database-specific strategies).
**Selection process**	8	Specify the methods used to decide whether a study met the inclusion criteria of the review, including how many reviewers screened each record and each report retrieved, whether they worked independently, and if applicable, details of automation tools used in the process.	Section 2.3. Study Selection (three independent reviewers; Rayyan QCRI; arbitration by senior reviewer).
**Data collection process**	9	Specify the methods used to collect data from reports, including how many reviewers collected data from each report, whether they worked independently, any processes for obtaining or confirming data from study investigators, and if applicable, details of automation tools used in the process.	Section 2.4. Data Extraction (standardized form; independent extraction; third reviewer verification).
**Data items**	10a	List and define all outcomes for which data were sought. Specify whether all results that were compatible with each outcome domain in each study were sought (e.g. for all	Section 2.1 (Outcomes in PICOS) and Section 2.4 (primary outcome: recurrent stroke; secondary outcomes: composite vascular

Section and Topic	Item #	Checklist item	Location where item is reported
		measures, time points, analyses), and if not, the methods used to decide which results to collect.	events, SAEs, any AEs, treatment discontinuation).
	10b	List and define all other variables for which data were sought (e.g. participant and intervention characteristics, funding sources). Describe any assumptions made about any missing or unclear information.	Section 2.4. Data Extraction (author, year, design, country, sample size, eligibility, interventions, timing of initiation, follow-up duration).
**Study risk of bias assessment**	11	Specify the methods used to assess risk of bias in the included studies, including details of the tool(s) used, how many reviewers assessed each study and whether they worked independently, and if applicable, details of automation tools used in the process.	Section 2.5. Risk of Bias Assessment (RoB 2 tool; two independent reviewers; discrepancies resolved with a third author).
**Effect measures**	12	Specify for each outcome the effect measure(s) (e.g. risk ratio, mean difference) used in the synthesis or presentation of results.	Section 2.6. Meta-Analysis (risk ratios [RR] with 95% CI).
**Synthesis methods**	13a	Describe the processes used to decide which studies were eligible for each synthesis (e.g. tabulating the study intervention characteristics and comparing against the planned groups for each synthesis (item #5)).	Section 2.1 (eligibility/strata) and Section 2.6 (pre-planned syntheses by stratum).
	13b	Describe any methods required to prepare the data for presentation or synthesis, such as handling of missing summary statistics, or data conversions.	Section 2.6. Meta-Analysis (continuity correction applied where appropriate, e.g., LoDoCo; see Results Section 3.4.3).
	13c	Describe any methods used to tabulate or visually display results of individual studies and syntheses.	Section 2.6 (forest plots for all outcomes; Figures 2–4); Table 1 and Table 3.
	13d	Describe any methods used to synthesize results and provide a rationale for the choice(s). If meta-analysis was performed, describe the model(s), method(s) to identify the presence and extent of statistical heterogeneity, and software package(s) used.	Section 2.6. Meta-Analysis (inverse-variance random-effects model; HKSJ confidence intervals; τ^2 by Paule–Mandel; I^2 and 95% prediction intervals; R v4.5.2, meta and metafor packages).
	13e	Describe any methods used to explore possible causes of heterogeneity among study results (e.g. subgroup analysis, meta-regression).	Section 2.6 (pre-planned subgroup by timing of colchicine initiation: ultra-acute, subacute, chronic; sensitivity analysis restricted to stroke/TIA-dedicated trials).
	13f	Describe any sensitivity analyses conducted to assess robustness of the synthesized results.	Section 2.6 (leave-one-out analyses; sensitivity restricted to stroke/TIA trials). Results in Sections 3.4.1–3.4.3.
**Reporting bias assessment**	14	Describe any methods used to assess risk of bias due to missing results in a synthesis (arising from reporting biases).	Section 2.7 (publication bias considered within the GRADE framework). Formal funnel plot/Egger test not performed due to small number of trials (<10).

Section and Topic	Item #	Checklist item	Location where item is reported
**Certainty assessment**	15	Describe any methods used to assess certainty (or confidence) in the body of evidence for an outcome.	Section 2.7. Assessment of the Certainty of the Evidence (GRADE framework: risk of bias, imprecision, indirectness, inconsistency, publication bias).
**RESULTS**			
**Study selection**	16a	Describe the results of the search and selection process, from the number of records identified in the search to the number of studies included in the review, ideally using a flow diagram.	Section 3.1. Study Selection (1,114 records; 5 included). Figure 1. PRISMA 2020 Flow chart.
	16b	Cite studies that might appear to meet the inclusion criteria, but which were excluded, and explain why they were excluded.	Section 3.1; Supplementary Table S3 (Reasons for exclusion of studies).
**Study characteristics**	17	Cite each included study and present its characteristics.	Section 3.2. Characteristics of the included studies; Table 1.
**Risk of bias in studies**	18	Present assessments of risk of bias for each included study.	Section 3.3. Risk of bias assessment; Table 2.
**Results of individual studies**	19	For all outcomes, present, for each study: (a) summary statistics for each group (where appropriate) and (b) an effect estimate and its precision (e.g. confidence/credible interval), ideally using structured tables or plots.	Sections 3.4.1–3.4.3; Figures 2, 3 and 4 (forest plots for recurrent stroke, composite vascular events, and SAEs).
**Results of syntheses**	20a	For each synthesis, briefly summarise the characteristics and risk of bias among contributing studies.	Sections 3.2 and 3.3 (study characteristics and RoB summary); Tables 1 and 2.
	20b	Present results of all statistical syntheses conducted. If meta-analysis was done, present for each the summary estimate and its precision (e.g. confidence/credible interval) and measures of statistical heterogeneity. If comparing groups, describe the direction of the effect.	Sections 3.4.1 (recurrent stroke RR 0.86, 95% CI 0.65–1.12; I^2 = 25.5%), 3.4.2 (vascular events RR 0.70, 95% CI 0.40–1.23; I^2 = 85.0%), 3.4.3 (SAEs RR 1.14, 95% CI 0.43–2.97; I^2 = 45.4%). Figures 2–4.
	20c	Present results of all investigations of possible causes of heterogeneity among study results.	Sections 3.4.1–3.4.2 (subgroup by stroke/TIA-dedicated vs. ASCVD trials; subgroup by timing of initiation).
	20d	Present results of all sensitivity analyses conducted to assess the robustness of the synthesized results.	Sections 3.4.1–3.4.3 (sensitivity restricted to stroke/TIA trials; leave-one-out analyses, e.g., exclusion of LoDoCo2 for vascular events).
**Reporting biases**	21	Present assessments of risk of bias due to missing results (arising from reporting biases) for each synthesis assessed.	Section 3.5 and Table 3 (publication bias considered within GRADE). Funnel plot/Egger test not performed (k<10).
**Certainty of evidence**	22	Present assessments of certainty (or confidence) in the body of evidence for each outcome assessed.	Section 3.5. Certainty of the Evidence (GRADE); Table 3

Section and Topic	Item #	Checklist item	Location where item is reported
			(moderate for recurrent stroke; low for vascular events and SAEs).
**DISCUSSION**			
**Discussion**	23a	Provide a general interpretation of the results in the context of other evidence.	Section 4. Discussion (paragraphs 1–5).
	23b	Discuss any limitations of the evidence included in the review.	Section 4.3. Strengths and limitations.
	23c	Discuss any limitations of the review processes used.	Section 4.3. Strengths and limitations.
	23d	Discuss implications of the results for practice, policy, and future research.	Section 4.2. Clinical implications; Section 4.3 (future research); Section 5. Conclusions.
**OTHER INFORMATION**			
**Registration and protocol**	24a	Provide registration information for the review, including register name and registration number, or state that the review was not registered.	Methods: the review was not prospectively registered in PROSPERO.
	24b	Indicate where the review protocol can be accessed, or state that a protocol was not prepared.	A formal a priori protocol was not prepared; the PICOS framework and pre-planned analyses are described in Sections 2.1 and 2.6.
	24c	Describe and explain any amendments to information provided at registration or in the protocol.	Not applicable (no registered protocol).
**Support**	25	Describe sources of financial or non-financial support for the review, and the role of the funders or sponsors in the review.	Funding statement (back matter): "This research received no external funding. The APC was funded by Universidad Señor de Sipán – Vicerrectorado de Investigación."
**Competing interests**	26	Declare any competing interests of review authors.	Conflicts of Interest statement (back matter): "The authors declare that they have no competing interests."
**Availability of data, code and other materials**	27	Report which of the following are publicly available and where they can be found: template data collection forms; data extracted from included studies; data used for all analyses; analytic code; any other materials used in the review.	Data Availability Statement (back matter): "Data are contained within the article." Supplementary Tables S1–S3 contain the PRISMA checklist, search strategies and reasons for exclusion.

From: Page MJ, McKenzie JE, Bossuyt PM, Boutron I, Hoffmann TC, Mulrow CD, et al. The PRISMA 2020 statement: an updated guideline for reporting systematic reviews. BMJ 2021;372:n71. doi: 10.1136/bmj.n71.

For more information, visit: http://www.prisma-statement.org/

Supplementary table S2. Search strategy for each database

ENGINE	STRATEGY	RESULTS
Pubmed	#1 = ("Cryptogenic Stroke" OR "Acute Ischemic Stroke" OR "Acute Ischemic Strokes" OR "Cryptogenic Ischemic Stroke" OR "Cryptogenic Ischemic Strokes" OR "Stroke on Waking Up" OR "Cryptogenic Embolism Cerebrovascular Accident" OR "Embolism Stroke" OR "Wake-up Stroke" OR "Stroke, Cryptogenic" OR "Ischemic Stroke, Cryptogenic" OR "Stroke, Cryptogenic Ischemic" OR "Stroke, Cryptogenic Embolism" OR "Stroke, Wake-up" OR "Stroke, Ischaemic" OR "Ischemic Strokes" OR "Ischemic Stroke" OR "Ischemic Stroke, Acute" OR "Stroke, Acute Ischemic" OR "Strokes on Waking Up" OR "Thrombotic Stroke")<br>#2 = (Colchicine OR "Colchicine Therapy")<br>#3= #1 AND #2	72
Scopus	#1 = TITLE-ABS-KEY ("Cryptogenic Stroke" OR "Acute Ischemic Stroke" OR "Acute Ischemic Strokes" OR "Cryptogenic Ischemic Stroke" OR "Cryptogenic Ischemic Strokes" OR "Stroke on Waking Up" OR "Cryptogenic Embolism Cerebrovascular Accident" OR "Embolism Stroke" OR "Wake-up Stroke" OR "Stroke, Cryptogenic" OR "Ischemic Stroke, Cryptogenic" OR "Stroke, Cryptogenic Ischemic" OR "Stroke, Cryptogenic Embolism" OR "Stroke, Wake-up" OR "Stroke, Ischaemic" OR "Ischemic Strokes" OR "Ischemic Stroke" OR "Ischemic Stroke, Acute" OR "Stroke, Acute Ischemic" OR "Strokes on Waking Up" OR "Thrombotic Stroke")<br>#2= TITLE-ABS-KEY(Colchicine OR "Colchicine Therapy")<br>#3= #1 AND #2	153
Web of Science	#1 = ("Cryptogenic Stroke" OR "Acute Ischemic Stroke" OR "Acute Ischemic Strokes" OR "Cryptogenic Ischemic Stroke" OR "Cryptogenic Ischemic Strokes" OR "Stroke on Waking Up" OR "Cryptogenic Embolism Cerebrovascular Accident" OR "Embolism Stroke" OR "Wake-up Stroke" OR "Stroke, Cryptogenic" OR "Ischemic Stroke, Cryptogenic" OR "Stroke, Cryptogenic Ischemic" OR "Stroke, Cryptogenic Embolism" OR "Stroke, Wake-up" OR "Stroke, Ischaemic" OR "Ischemic Strokes" OR "Ischemic Stroke" OR "Ischemic Stroke, Acute" OR "Stroke, Acute Ischemic" OR "Strokes on Waking Up" OR "Thrombotic Stroke")<br>#2 = (Colchicine OR "Colchicine Therapy")<br>#3= #1 AND #2	66
Embase	#1= ('acute ischemic stroke'/exp OR 'acute ischemic stroke' OR 'cerebrovascular accident')<br>#2= ('Colchicine therapy' OR 'colchicine')<br>#3= #1 AND #2	823

Supplementary table S3. Reason for exclusion of studies

Title	Author	Reason for exclusion
Long-term trials of colchicine for secondary prevention of vascular events: a meta-analysis	Michelle Samuel , Colin Berry , Marie-Pierre Dubé, Wolfgang Koenig , José López-Sendón , Aldo Pietro Maggioni, Fausto J. Pinto, François Roubille , and Jean-Claude Tardif	Other design
CONVINCE - (COlchicine for preventioN of Vascular Inflammation in Non-CardioEmbolic Stroke) - a Randomised Clinical Trial of Low-dose Colchicine for Secondary Prevention After Stroke	Health Research Board I, Irish Heart F, University of L, et al	Other population
Cardiovascular Benefit of Colchicine in Relation to Baseline Risk: A Secondary Analysis of the LoDoCo2 Trial	Niekbachsh Mohammadnia , Britta E Wesselink , Willem A Bax , Stefan M Nidorf , Arend Mosterd , Aernoud T L Fiolet , Aysun Cetinyurek-Yavuz , Peter L Thompson, Shrikant I Bangdiwala , John W Eikelboom , Jan H Cornel, Saloua El Messaoudi	Other population
